# Humidification and heating of inhaled gas in patients with artificial airway. A narrative review

**DOI:** 10.5935/0103-507X.20180015

**Published:** 2018

**Authors:** Gustavo Adrián Plotnikow, Matias Accoce, Emiliano Navarro, Norberto Tiribelli

**Affiliations:** 1 Capítulo de Kinesiología Intensivista, Sociedad Argentina de Terapia Intensiva - Buenos Aires, Argentina.; 2 Sanatorio Anchorena - Buenos Aires, Argentina.; 3 Sanatorio Anchorena San Martín -Buenos Aires, Argentina.; 4 Hospital Durand - Buenos Aires, Argentina.; 5 Complejo Médico de la Policía Federal Argentina Churruca Visca - Buenos Aires, Argentina.

**Keywords:** Humidification systems, Mechanical ventilation, Airway management

## Abstract

Instrumentation of the airways in critical patients (endotracheal tube or tracheostomy cannula) prevents them from performing their function of humidify and heating the inhaled gas. In addition, the administration of cold and dry medical gases and the high flows that patients experience during invasive and non-invasive mechanical ventilation generate an even worse condition. For this reason, a device for gas conditioning is needed, even in short-term treatments, to avoid potential damage to the structure and function of the respiratory epithelium. In the field of intensive therapy, the use of heat and moisture exchangers is common for this purpose, as is the use of active humidification systems. Acquiring knowledge about technical specifications and the advantages and disadvantages of each device is needed for proper use since the conditioning of inspired gases is a key intervention in patients with artificial airway and has become routine care. Incorrect selection or inappropriate configuration of a device can have a negative impact on clinical outcomes. The members of the *Capítulo de Kinesiología Intensivista* of the *Sociedad Argentina de Terapia Intensiva* conducted a narrative review aiming to show the available evidence regarding conditioning of inhaled gas in patients with artificial airways, going into detail on concepts related to the working principles of each one.

## INTRODUCTION

The conditioning of medical gases administered to patients has become a routine healthcare procedure. Under normal conditions, the upper airway and respiratory tract are responsible for humidify and heating inspired air, a process defined as conditioning of inhaled gas.^(1,2)^ This process is critical for optimally conditioning the gas and avoiding potential damage to the structure and function of the respiratory epithelium.^(3-6)^

Instrumentation of the airways (endotracheal tube or tracheostomy cannula) prevents the conditioning of inspired gas. In addition, the administration of cold and dry medical gases and the high flows that patients experience with invasive (iMV) or non-invasive mechanical ventilation (NIV) lead to an even worse condition.^(7)^ For this reason, the use of an external device for conditioning the delivered gases is required, even in short-term treatments.

## METHODS

A literature search was performed by the *Capítulo de Kinesiología Intensivista* of the *Sociedad Argentina de Terapia Intensiva* (SATI) to develop the present narrative review. The search was performed using the following databases: LILACS, MEDLINE, Cochrane library and SciELO, and the following terms and combinations of words: *"randomized controlled trial" OR "controlled clinical trial" OR "trial" OR "groups" AND "humidifiers" AND "heat and moisture exchangers" OR "heat and moisture exchangers filters" AND "heated humidifiers" OR "heated humidified" AND "mechanical ventilation" AND "noninvasive ventilation" AND "spontaneous breathing".* The most relevant articles were chosen according to the authors' criteria.

### Concept of humidity

**Figure 1:**
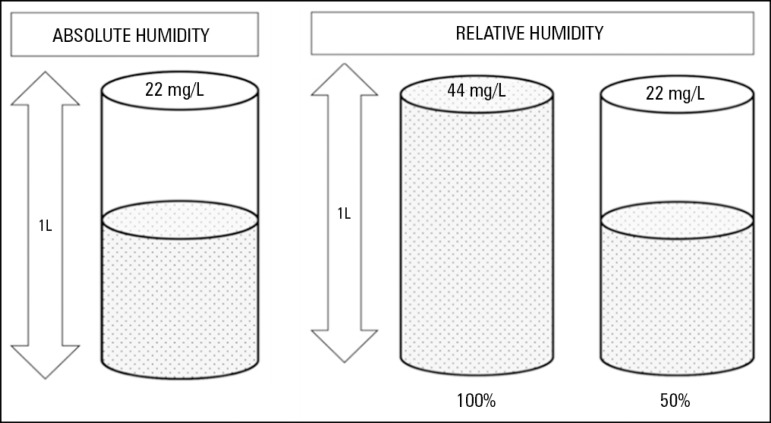
Graphical description of absolute and relative humidity.

Humidity is the amount of water vapor contained in a gas and is, in general, characterized in terms of absolute or relative humidity.^(8-10)^ The temperature of the gas is very important because its water vapor content depends on gas temperature. Relative humidity is the percentage (%) of water vapor contained in a gas relative to its maximum carrying capacity. Absolute humidity is the total amount of water vapor contained in a gas, expressed in milligrams of water suspended in liters of gas (mg/L). Absolute humidity is directly related to the gas temperature and is important in terms of humidification since at low temperatures, the relative humidity can be 100%, while the absolute humidity can be far below the recommended value^(8,9)^ (Figure 1 and Table 1).

**Table 1:** Relationships between the gas temperature, absolute humidity and water vapor pressure

Gas temperature (ºC)	Absolute humidity (mg/L)	Water vapor pressure (mmHg)
0	4.85	4.6
5	6.8	6.5
10	9.4	9.2
15	12.8	12.8
20	17.3	17.5
25	23.0	23.7
30	30.4	31.7
32	33.8	35.5
34	37.6	39.8
36	41.7	44.4
37	43.9	46.9*
38	46.2	49.5
40	51.1	55.1
42	56.5	61.3
44	62.5	68.1

* Ideal condition for gas conditioning, limit of isothermal saturation.

### Physiology of the upper airway. Role in gas conditioning

The conditioning of inspired air is the process by which a gas is warmed and moisturized during its passage through the airways to reach the alveolar level under optimal conditions.^(5)^

Although the gas acquires temperature and humidity during its passage through the airway, the main area where the heating of inspired air occurs is the nose.^(5,11-13)^ The temperature of nasal mucosa is approximately 32ºC, and although the contact time between inspired air and nasal mucosa is short, this time is sufficient to transfer heat. In addition, the nose has a great potential to regulate blood perfusion and thus counterbalance the loss of heat during inspiration. Moreover, the air circulates through a narrow conduit generating turbulent flow that allows optimizations in heating, humidification and filtering.^(12,13)^ During expiration, the humidity of exhaled air is partially preserved by condensation on the mucosa due to differences in temperature. Approximately 25% of the heat and humidity is recovered during exhalation.^(11)^

Since the temperature of the inspired air increases during its passage through the airway, at the level of the alveolar-capillary interface, it is at body temperature (37ºC), with 100% relative humidity and 44mg/L absolute humidity. The point where the gas acquires these conditions is known as the isothermal saturation limit; this limit is usually close to the 4^th^ or 5^th^ bronchial generation. It is critical to reach the isothermal saturation limit to avoid damage to the mucosa and the ciliary epithelium.^(7)^

**Figure 2:**
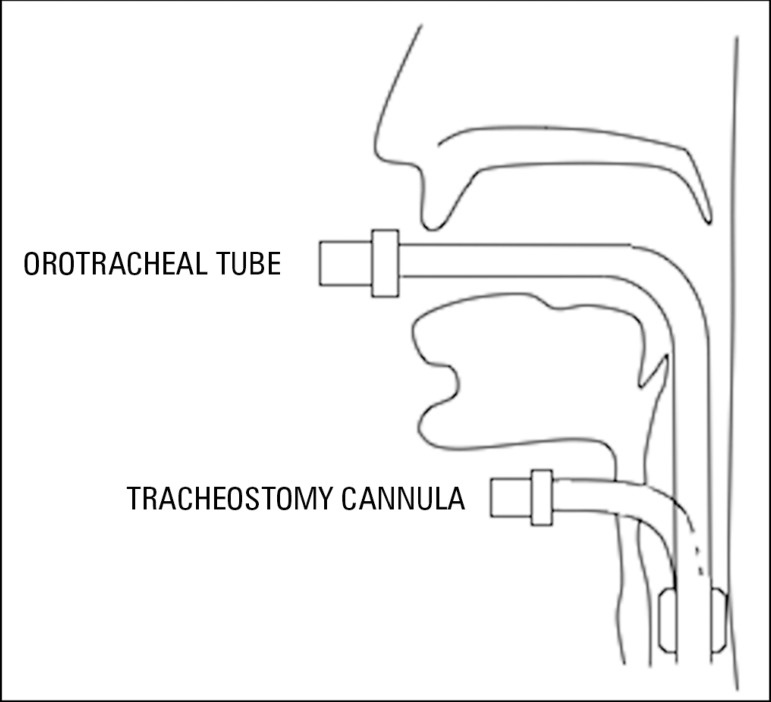
Bypass of the airway based on the type of artificial airway.

The presence of an artificial airway prevents inspired air from contacting the mucosa of the upper airway affecting gas conditioning (Figure 2). Moreover, if medical gases are administered, there is a risk of generating alterations in clearance and mucosa^(5,8)^ because these medical gases are cold and dry.^(14)^

Two types of devices for conditioning inspired gases in the presence or absence of an artificial airway are available: heat and moisture exchangers (HME) and active humidifiers.^(15)^

Regardless of which device is chosen, it should infallibly meet the minimum requirements to replace the function of the upper airway, which, according the American Association for Respiratory Care, are:^(9)^

- 30mg/L absolute humidity, 34ºC and 100% relative humidity for HME.
- Between 33 and 44mg/L absolute humidity; between 34ºC and 41ºC; 100% relative humidity for active humidifiers.

## PASSIVE HUMIDIFIERS

### Classification

The working principle for these devices is based on their capacity to retain heat and humidity during expiration and to deliver at least 70% of them to the inhaled gas during subsequent inspiration. This "passive" function can be achieved by different mechanisms, and the classification of these devices is based on their mechanisms.^(16-22)^

### a. Heat and moisture exchangers

**Figure 3:**
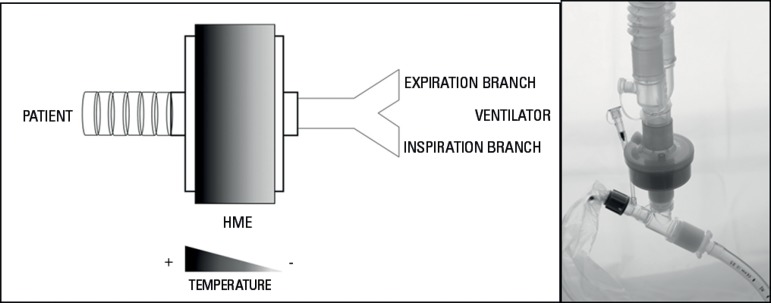
Schematic description of the working principle of a heat and moisture exchanger and placement in the ventilator circuit.

Heat and moisture exchangers are simple condensers constructed with elements made of disposable foam, synthetic fiber or paper, with a significant surface area that can generate an effective temperature gradient through the device delivering heat on each inspiration. From the field of filtration, hydrophobic HMEs were made to repel the humidity and to retain the heat of the expired gas, delivering it in the next inspiration (Figure 3).^(8,16,23,24)^

### b. Hygroscopic condenser humidifiers (HCHs) or hygroscopic heat and moisture exchangers (HHMEs)

In contrast with HMEs, HCHs or HHMEs are devices made with synthetic fiber coated by a hygroscopic chemical product (calcium chloride or lithium chloride), which absorbs expired water vapor and delivers it to the inspired gas, optimizing the delivery of humidity. The synthetic fiber also helps to decrease the accumulation of condensation in a device-dependent position.^(8,17,18)^

### c. Heat and moisture exchangers with filter

These devices are made with materials needed for moisturizing and heating based on their working principle (hydrophobic or hygroscopic); in addition, they contain an electrostatic filter. Filters are flat layers of fiber material (modacrylic or propylene) that act as a barrier to the gas flow; at the same time, the filtration performance is optimized by applying material with electrostatic charge.^(25)^

The available types of filters are: heat and moisture exchanger filters (HMEFs), hygroscopic condenser humidifier filters (HCHFs) or hygroscopic heat and moisture exchanger filters (HHMEFs).^(18)^

### d. Combined heat and moisture exchangers

Hygroscopic and hydrophobic elements are used combined to create a combined HME. The performance in terms of absolute humidity is better in HCHs than in HMEs. However, the performances are similar between HCHs and combined HMEs.^(24,26)^

## PASSIVE HUMIDIFIERS - SPECIAL CONSIDERATIONS

### Dead space

**Figure 4:**
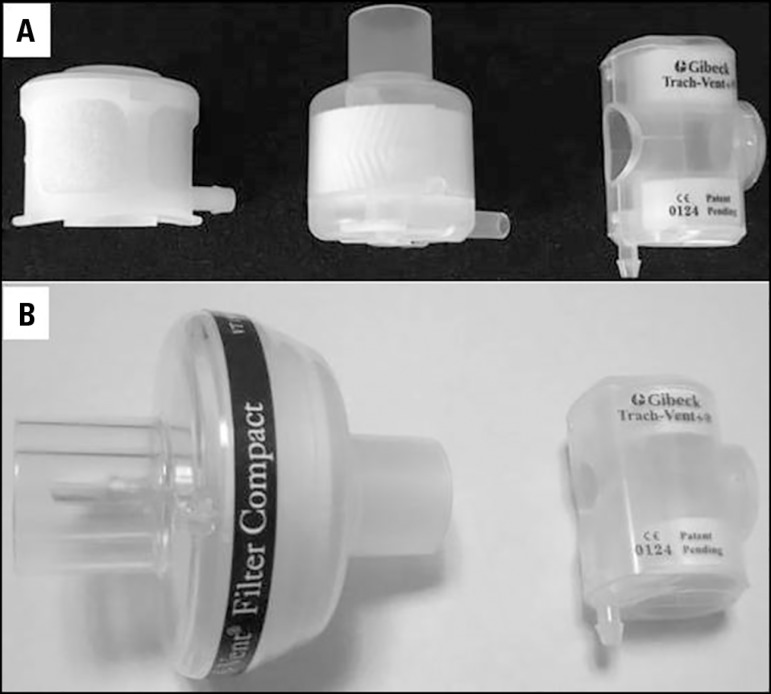
**A)** Heat and moisture exchangers for patients with artificial airways in spontaneous ventilation (particularly in tracheotomized patients). **B)** Graphic comparison between volumes of a conventional heat and moisture exchanger for use in patients with artificial airway in mechanical ventilation and one for use in tracheotomized patients with spontaneous ventilation.

The working principle of passive humidifiers implies that a higher volume of condenser material will yield better device performance (Figure 4). For this reason, the 'ideal' dead space for a humidifier is approximately 50mL.^(18)^

This dead space does not represent a disadvantage for patients with iMV because the dead space can be compensated for in the programming of the ventilator. However, in patients with artificial airways, without the requirement of iMV, the increase in the ventilatory minute volume as a compensatory mechanism for the dead space could generate a hard-to-tolerate load in patients with low ventilatory reserve. Therefore, passive humidifiers with 'small volume' were developed (Figure 4). Although they can be more 'tolerable', they have low humidification capacity that worsens when the tidal volume (V_T_) increases and with supplementary O_2_.^(27-30)^

The instrumental dead space added by the passives humidifiers during iMV becomes relevant when the pathology requires strategies for lung protection (low V_T_). Studies conducted by Prat^(31)^ and Hinkson^(32)^ account for this, demonstrating significant changes in arterial carbon dioxide partial pressure (PaCO_2_) (and in pH) under these circumstances.

### Resistance

Although the resistance of an unused device could be considered negligible (5cmH_2_O/L/sec assessed as 'dry": *International Standards Organization Draft International Standard* 9360-2),^(33)^ resistance can vary under changes in the conditions (presence of condensation, impaction due to secretions, changes in ventilatory parameters, increases in the V_T_ and the flow). Although some studies^(18,34,35)^ have shown that the presence of 'humidity' in a device does not lead to significant changes in resistance, excessive condensation or impaction (due to secretions or blood) can alter it.

## PASSIVE HUMIDIFIERS

### Implementation and monitoring

Due to the working principle of a passive humidifier, it should always be placed before the 'Y' part of the circuit, allowing the device to contact the inspired and expired air during each ventilation cycle (Figure 3), which makes the passive humidifier part of the instrumental dead space.^(19)^

### Volume of the heat and moisture exchanger and its relation to the humidification capacity

Several factors affect the delivery of humidity to the patient: internal volume and absolute humidity provided are 2 significant variables to consider.

**Figure 5:**
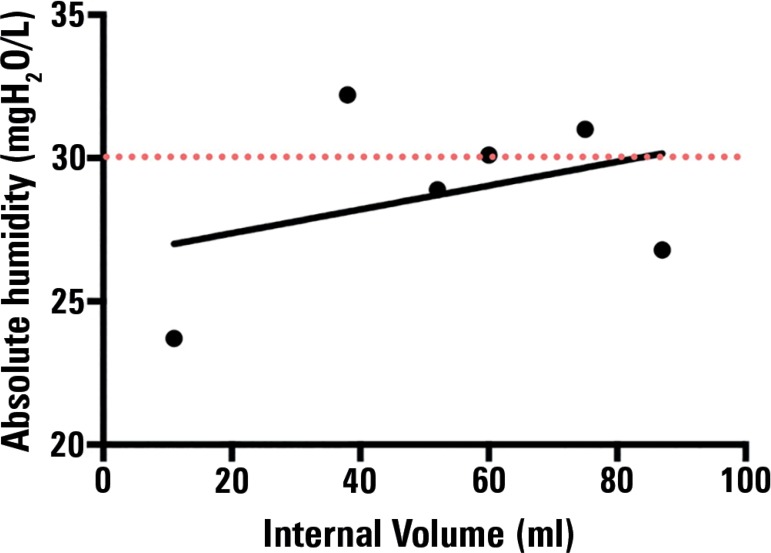
Correlation between internal volume and humidity delivered in Eckerbom's study. The programming of the ventilator corresponds to "Setting II" of the study (tidal volume of 500 -mL, respiratory frequency of 20 breaths per minute). Pearson's correlation coefficient was used for the statistical analysis.

Source: Based on data from the study: Eckerbom B, Lindholm CE. Performance evaluation of six heat and moisture exchangers according to the Draft International Standard (ISO/DIS 9360). Acta Anaesthesiol Scand. 1990;34(5):404-9.^(16)^

In a bench study, Eckerbom et al.^(16)^ related the dead space of HME with the absolute humidity delivered, reporting a poor correlation between both variables. However, only 6 devices were assessed; some of them were bacterial filters, so the results should be considered with caution. To analyze the results provided by this study in more detail, we drafted a scatter plot (Figure 5) with the abovementioned variables. This plot shows a poor correlation between these variables (r = 0.36; p = 0.47).

**Figure 6:**
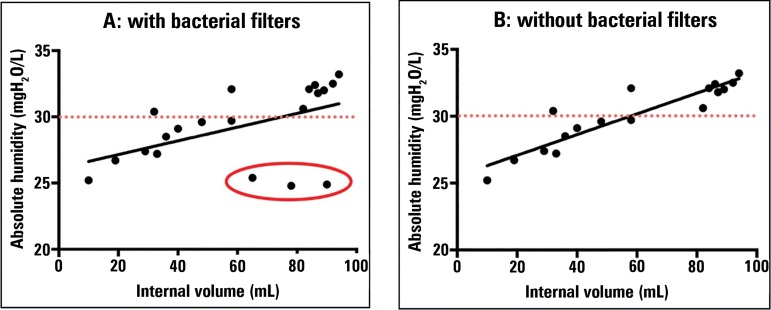
Correlation between the internal volume of the heat and moisture exchanger and the absolute humidity delivered. The programming of the ventilator corresponds to "Setting I" (tidal volume of 500mL, respiratory frequency of 20 breaths per minute). In "B", the same analysis as in "A" was conducted, but excluding the 3 bacterial filters (Pall, Intertech HEPA, Intersurgical). Pearson's correlation coefficient was used for the statistical analysis.

Source: Based on data from the study: Branson R, Davis K Jr. Evaluation of 21 passive humidifiers according to the ISO 9360 standard: moisture output, dead space, and flow resistance. Respir Care. 1996;41:736-43.^(18)^

Branson et al.^(18)^ tried to replicate the abovementioned study, with the same ISO standards but with more devices. Branson et al.^(18)^ showed that as the dead space increases, the delivered absolute humidity also increases. To further explore these results, we drafted correlation graphs using data provided by Branson et al.^(18)^ (Figure 6). Figure 6A shows poor correlation (r = 0.5; p = 0.01) between the 2 variables. However, when analyzing the devices used in detail, we found that 3 bacterial filters were included (marked with a red circle). When the analysis was repeated but excluding the results related to these 3 devices, we found an excellent correlation (Figure 6B; r = 0.91; p < 0.0001).

The ratio between the delivered absolute humidity and the dead space in mL is an efficacy measure described in the literature.^(18)^ Devices with a higher ratio are considered more effective, that is, they provide better delivery of absolute humidity per unit of internal volume. Humidifiers reaching the recommended limit of 30 mg/L^9^ had a dead space of approximately 60mL (Figure 6A and 6B).

Therefore, we can assume that the device volume influences its performance and that the volume should be taken into account when selecting an HME.

### Minute volume and performance of heat and moisture exchangers

The humidification performance of an HME decreases when minute volume increases.^(8,16,18-21)^ Some studies found that the absolute humidity delivered decreased as the V_T_ increased,^(8,16)^ whereas only Unal et al.^(21)^ found that the flow was a variable influencing the performance. The inconsistencies found between studies could be due to the use of different laboratory models (hygrometric or gravimetric methods). However, in 2014, Lellouche et al.^(36)^ investigated the impact of minute ventilation (10 *versus* 20L/minute) and the room temperature on the new generation of HMEs, reporting no significant effects on the performance of these devices [absolute humidity delivered with a minute volume > 10L/minute was 30.9mg/L]. For this reason, these authors recommend not taking into account the minute ventilation as a limitation of the new generation of humidifiers. Since the minute volume is composed of the respiratory frequency and the V_T_, it might be worth investigating this issue thoroughly. The increase of the V_T_ in the study was a modest 150mL (500 to 650mL, which means 30%), whereas the increase in respiratory frequency was greater, from 20 to 30 resp/minute (50%). The question becomes one of which of these 2 components of the minute volume [V_T_ or respiratory frequency] has a greater effect on a humidifier performance.

Two studies compared^(16,18)^ the performance in terms of humidification of the HMEs using methods and test conditions based on the ISO 9360:1992 standard. In both studies, a more significant effect on performance was observed when changing the V_T_ from 500mL to 1000mL (increase of 100%) compared with changing the respiratory frequency from 10 to 20 resp/minute (increase of 100%), which suggests that V_T_ has a primary effect.^(16,18)^ Nevertheless, due to the unusual use of a V_T_ of 1000 mL, the results should be considered with caution.

### Time of contact with the air

Eckerbom et al.^(16)^ assessed the time of contact of the inspired gas, and the authors did not find significant differences in delivered absolute humidity when comparing 1 second *versus* 2 seconds. Branson et al.^(18)^ suggested that the expiratory flow could be a variable to consider since as the expiratory flow increases, the time of contact between the gas and the HME decreases along with the absolute humidity delivered.

## PASSIVE HUMIDIFIERS - CHANGE

Although manufacturers provide general specifications for the changing of these devices, we could consider an 'ideal' recommendation that the change is performed:

- Due to excessive condensation that increases the resistance.
- Due to visible impaction with secretions or blood.
- Each 48 hours in patients with chronic obstructive pulmonary disease.
- Each 96 hours and up to 1 week in the remaining patients.^(37-39)^

## ACTIVE HUMIDIFIERS (HEATED HUMIDIFIERS)

Active humidifiers are devices composed of an electric heater on which is placed a plastic casing with a metallic base in which sterile water is stored. When the base is warmed, the water temperature increases by convection. Some active humidifiers are self-regulated by a mechanism consisting of a heating wire (heated-wire breathing circuit) that keeps the gas temperature constant during its passage through the circuit and a wire with two temperature sensors connected to the exit of the heater (distal) and to a part of the circuit (close to the patient) to control the system temperature.

It should be taken into account that while heated-wire breathing circuit keep the temperature stable throughout its route and decrease the condensation, the lack of control exposes the patient to risks such as a higher incidence of occlusion of the artificial airway.^(40)^

## TYPES OF ACTIVE HUMIDIFIERS

### Bubble humidifiers

The flow of entering air is forced to pass through a tube that leads it to the lower part of the casing. The gas exiting from the distal end of the tube (under the water surface) form bubbles that gain humidity and temperature as they rise to the water surface. The amount of water in the container and the flow rate are factors affecting the humidity content of the gas. Simply expressed, if the water column in the container is taller, the gas-water interface will increase. Inversely, as the circulating flow increases, the device performance drops. These types of devices are no longer used.^(41)^

### Pass-over humidifiers

The air flow passes over the water surface, which is hot, and the circulating gas gains heat and humidity from the gas-water interface formed.^(41)^ In comparison with the previously described device, pass-over humidifiers have lower resistance. It is important to consider that the water temperature in the enclosure will be a determining factor for humidity.^(42,43)^

A variant of the pass-over humidifier is the 'wick' humidifier, in which a porous membrane that absorbs humidity (such as blotting paper) is dipped into water surrounded by the heating element, keeping the membrane constantly saturated with water vapor. The dry gas enters the chamber, makes contact with the wick and is loaded with more water vapor than in the basic system because the gas-liquid interface is larger.

Another variant is the hydrophobic membrane humidifier, where dry gas passes through the membrane, and, due to the membrane properties, only water vapor passes through. The dry gas is loaded with water vapor and exits the chamber.

## ACTIVE HUMIDIFIERS - IMPLEMENTATION AND MONITORING

### Assembly

Active humidifiers are placed in-line in the inspiration branch of the respirator. The circuit exiting the inspiration valve is connected with the entry hole of the plastic casing, which should be always filled to the level indicated by the manufacturer. Subsequently, a second section of the circuit (of standard length) is connected with the exit hole of the casing and to the "Y" of the circuit responsible for delivering gases to the patient. 

With this type of humidification device, it is necessary to use water traps, reservoirs with unidirectional circulation systems that allow the deposition of surplus condensation without leaking air. Among other problems, the excessive accumulation of water in the circuit can lead to auto-triggering, misreading of the ventilator monitoring or even draining of contaminated material into the patient's airway.

### Precautions and monitoring

- Observe that the tubing drains the water downwards and not toward the artificial airway or the ventilator.
- Place the water traps correctly to receive drained water.
- Frequently monitor the active humidifier device (water level, temperature level, check the presence of condensation).
- Never fill above the recommended level.
- Do not drain the condensation toward the humidifier chamber.
- Comply with the manufacturer's specifications.

## SPECIAL HUMIDIFIERS

### Humidifiers for aerosol therapy

The HME for aerosol therapy Gibeck Humid-Flo^®^ is an HME that has the property of remaining in-line during the application of aerosols in mechanical ventilation, avoiding the opening of the circuit and potentially reducing the contamination of tubing and the exposure of healthcare staff to contaminated gases.

This type of humidifier has 2 modes: "HME and AEROSOL", which can be selected by rotating the central axis of the device. By choosing HME, the device acts as a conventional passive humidifier. In AEROSOL mode, the humidifier part is skipped; therefore, the aerosol goes directly to the patient without coming into contact with the humidifier material. However, the absolute humidity delivered with this device is at least questionable since it delivers 30.4mg/L, but with a V_T_ of 1000mL. 

Recently, in a bench study, Ari et al.^(44)^ assessed the performance of several HMEs to be used in combination with aerosol therapy. Those authors did not find significant differences in drug (albuterol) deposition in the artificial lung without HME (control) *versus* with HME, using a moisturized circuit (simulating the actual gas exhalation of a patient). Although the study was only laboratory-based, the HME modified for aerosol therapy seemed to not interfere with drug deposition.

### Heat and moisture exchanger-Booster

The HME-Booster^®^ is a type of hybrid active humidifier consisting of a passive humidification system and a 'T' connector containing a self-regulated heater and a small-volume chamber for continuous infusion of distilled water (consuming approximately 250mL every 3 days). The upper part of the device has a hydrophobic membrane that only allows the passage of water when it has evaporated toward the tubing. Therefore, the humidity from the water evaporated on the membrane is aggregated during inspiration, improving the gas conditions by approximately 3 - 4mg/L (depending on the V_T_, I:E ratio and the type of HME used).^(45)^ During the expiratory phase, heat and humidity are retained by the passive properties of the humidification system. The dead space of the device is approximately 9mL.

Advantages: easy to use, eliminates excessive condensation in the tubing, could be useful for hypothermic patients and has a bacterial filter (active humidifiers do not have a bacterial filter).

Disadvantages: it does not include temperature monitoring, increases resistance and requires a source of electrical energy (potential risk of burns and/or electric shocks).^(46)^

### Active hygroscopic heat and moisture exchangers

This device consists of an HHME in a heated casing in the shape of a cone with a radiator and an electronic controller. There is a line between the device and the water source, another line between the radiator and the controller and a third heating line between the device and the controller. The casing contains a paper element that provides a surface for humidity transfer to the gas. A water source drips continuously on the paper element, and the casing heat makes the liquid evaporate, increasing the humidity of the inhaled gas. The device is electronically controlled through a water and temperature control unit used to program the volume per minute of the patient.^(47)^

Some of the proposed advantages are eliminating condensation from the inspiration branch and avoiding the use of water traps. In addition, if the water source is depleted, this device continues working like an HHME (providing approximately 28 - 31mg/L absolute humidity). The active HHME provides temperatures of 36 - 38ºC and 90 - 95% relative humidity. The absolute humidity provided by the device is similar to the HME or active humidifiers but with lower water use and less condensation. The dead space of the device is 73mL, and the resistance is 1.7cmH_2_O/L/s.^(47)^

The disadvantages of this product are the possibility of skin burns and greater dead space compared with a heated humidifier or only an HHME.

## PASSIVE AND ACTIVE HUMIDIFIERS

### Advantages and disadvantages

**Figure 7:**
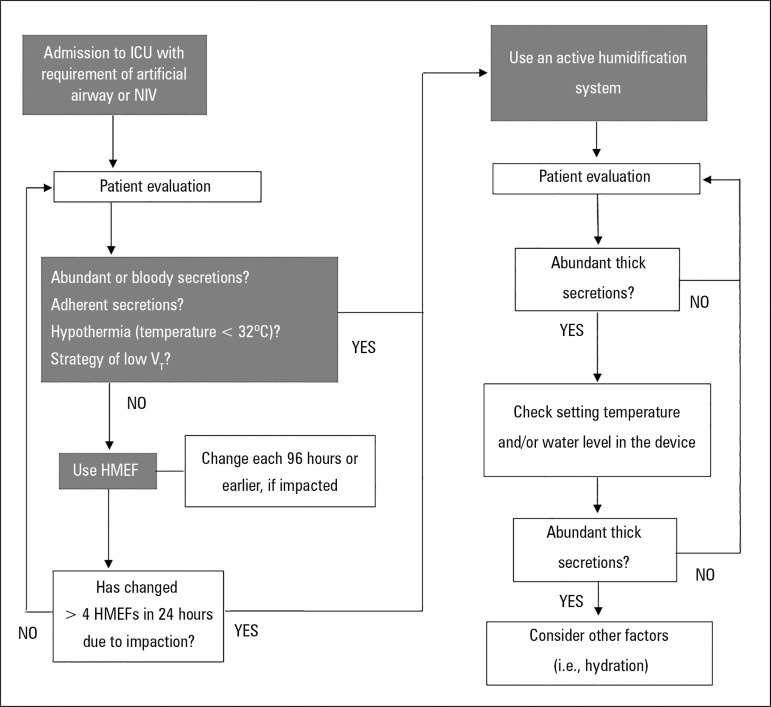
Algorithm for the selection of the humidification device. ICU - intensive care unit; AA - artificial airway; NIV - non-invasive mechanical ventilation; VT - tidal volume; HMEF - heat and moisture exchanger with filter.

Source: Adapted from: Branson RD. Humidification for patients with artificial airways. Respir Care. 1999;44(6):630-41.^(50)^

Taking into account the humidification capacity and the advantages and disadvantages, both passive and active humidifiers are suitable for the conditioning of inhaled gas (Table 2).^(48,49)^ Although there are some data that favor the use of HMEF (or HCHF) over active humidifiers for the prevention of pneumonia associated with mechanical ventilation, the choice of the device should not be based only on infection control.^(9)^ An algorithm for the selection of the humidification device is proposed in figure 7.

**Table 2:** Advantages and disadvantages of active and passive humidification systems

	**Advantages**	**Disadvantages**
**Active humidifiers**	They do not have contraindications. As they are placed in the inspiratory branch, they do not add instrumental dead space. If used correctly, they do not increase the resistance. They have alarm systems. They are more efficient and can deliver accurate temperatures.	Possibility of electrocution of the patient and the operator. They expose the airway lesion to excessive temperature. The presence of water in the circuit can limit the airflow. Condensation generates changes in the airway pressure and can lead to asynchronies. Colonization of the circuit. They require more monitoring to ensure correct performance.
	**Advantages**	**Disadvantages**
**Passive humidifiers**	Their use is simple. They do not have risks related to the treatment equipment. They are light in weight. They are cheaper. They are easily available in the intensive care unit.	They are contraindicated in some pathologies or clinical conditions. Because they are placed before the 'Y' in the circuit, they increase the instrumental dead space. They increase the resistance (negligible in most cases). They have lower performance or efficiency compared with an active humidifier. They should be retired to be able to apply aerosol therapy (except Gibeck Humid-Flo HME^®^).

**Figure 8:**
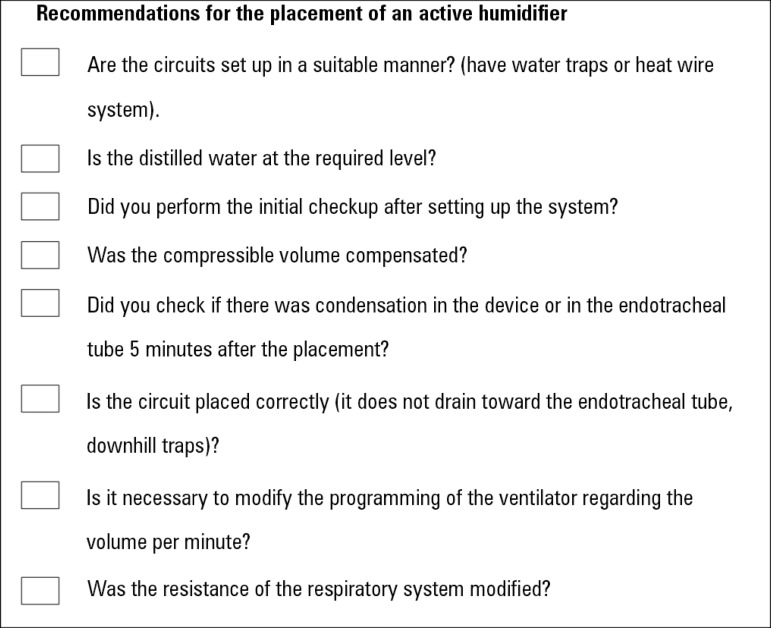
Recommendations to take into account for placement of an active humidifier.

**Figure 9:**
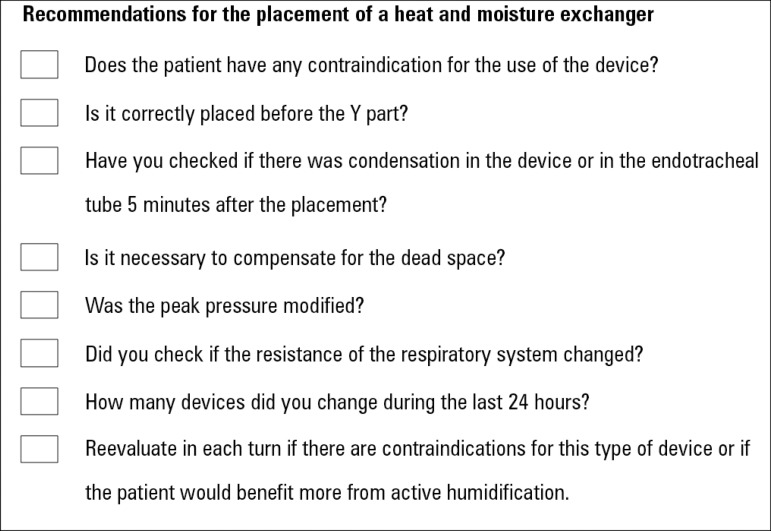
Recommendations to take into account for the placement of a heat and moisture exchanger.

To simplify the checks that should be performed when choosing a humidification system, we have provided this short list containing the main points to be considered (Figures 8 and 9).

When deciding which humidification system should be used, one should take into account the different clinical scenarios since each device features particular properties that can affect the clinical situation of the patient (Table 3).^(9,27-29,31,32,42,43,51-61)^ Given below are some clinical situations that should be considered:

- Acute Respiratory Distress Syndrome: The ventilatory strategy of lung protection uses low V_T_ (4 to 6mL/kg predicted body weight), which implies the risk of generating hypercapnia due to hypoventilation. It becomes rational to decrease the instrumental dead space as much as possible to counteract this adverse effect. An easy strategy to implement is the use of active humidifiers in these situations.^(31,32,53,54)^
- Ventilator associated-pneumonia (VAP): Decreasing the VAP rate is achieved by the adoption of a series of measures; humidification systems are not part of these measures (CDC 2014). For this reason, it is recommended to not choose the humidification device based on infection control.^(51,52)^
- NIV: The high flows achieved by the devices and leaks predispose them to heat and moisture loss during the respiratory cycle. Humidification increases the chances of success in the application of NIV since it is related to the capacity of managing respiratory secretions and increases the feeling of comfort. The device of choice for gas conditioning in NIV would be the active humidifier, with continuous flow ventilators and a one-branch circuit. In microprocessor-controlled ventilators with a two-branch circuit, this recommendation could be debated.^(60,61)^
- Spontaneous ventilation and artificial airways: In patients with tracheostomy (without iMV), it is advised to use the corresponding HME (considering their limitations) as the first-line treatment. In case it is contraindicated, then active humidification systems with a water temperature in the casing not below 53ºC should be used.^(42,43)^

**Table 3:** Humidification systems: clinical considerations

VAP	It is recommended not to select a humidification device based on its relationship with infection control
ARDS	The use of an active humidifier is suggested since it maintains its performance despite the ventilatory strategy used. It allows the optimization of the effective alveolar ventilation and decreases the VT and the instrumental dead space.
COPD	The use of an active humidifier is suggested since it does not increase the instrumental dead space or the ventilatory demand, without the risk of generating auto-PEEP. If using HME, change every 48 hours
Burned	The use of an active humidifier is suggested in patients with large body surfaces affected and inhalatory lesions due to its stable performance
Hypothermia	The use of an active humidifier is suggested in patients under a hypothermia strategy since they have demonstrated a better performance than HMEs and active HMEs
BPF	The use of an active humidifier is suggested in cases with an exhaled volume below 70% of the inhaled volume
NIV	Patients requiring ventilatory support for more than 1 hour are candidates for the use of a humidification device. The recommended device is an active humidifier with continuous flow ventilators with a one-branch circuit. In microprocessed ventilators with two-branch circuits, this recommendation is questionable.
Disconnection from MV	In patients with a problem in PaCO_2_ due their pathology or who present significant muscle weakness, the use of an active humidifier is recommended
Spontaneous ventilation	The use of a humidifier bottles as a conditioning system for the inhaled gases of a patient with artificial airways is discouraged

VAP - ventilator-associated pneumonia; ARDS - acute respiratory distress syndrome; VT - tidal volume; COPD - chronic obstructive pulmonary disease; PEEP - positive end expiratory pressure; HME - heat and moisture exchanger; BPF - bronchopleural fistula; NIV - non-invasive mechanical ventilation; MV - mechanical ventilation; PaCO_2_ - carbon dioxide partial pressure.

## CONCLUSIONS

Knowledge of the technical specifications and the advantages and disadvantages of each of the humidification devices is essential for professionals who provide care to patients in the intensive care unit. The conditioning of inspired gases is a key intervention in patients with artificial airways and has become a routine care strategy. The incorrect selection of the device or an inappropriate configuration can negatively affect the clinical outcome by damaging the airway mucosa, increasing respiratory effort or prolonging invasive mechanical ventilation.
